# miR-155 Inhibits Mouse Osteoblast Differentiation by Suppressing SMAD5 Expression

**DOI:** 10.1155/2017/1893520

**Published:** 2017-04-03

**Authors:** Yue Gu, Lianjun Ma, Lei Song, Xiaoping Li, Dong Chen, Xiaoxue Bai

**Affiliations:** ^1^Department of Respiratory Medicine, The First Hospital of Jilin University, 71 Xinmin Street, Changchun, China; ^2^Endoscopy Center, The China-Japan Hospital of Jilin University, 146 Xiantai Street, Changchun, China; ^3^Department of Pediatrics, The First Hospital of Jilin University, 71 Xinmin Street, Changchun, China; ^4^Cadre's Ward, The First Hospital of Jilin University, 71 Xinmin Street, Changchun, China

## Abstract

Osteogenesis from preosteoblasts is important for bone tissue engineering. MicroRNAs are a class of endogenous small RNA molecules that potentially modulate osteogenesis. In this study, we found that miR-155 expression was downregulated in a time-dependent manner in cells of the preosteoblast cell line MC3T3-E1 after osteogenic induction using bone morphogenetic protein 2 (BMP2). Transfection with miR-155 decreased alkaline phosphatase (ALP) activity, ALP expression, and the staining intensity of Alizarin Red in MC3T3-E1 cells treated with BMP2, whereas treatment with miR-155 inhibitor promoted BMP2-induced osteoblast differentiation. The luciferase assay confirmed that miR-155 can bind to the 3′ untranslated region of SMAD5 mRNA. miR-155 transfection significantly decreased the expression of SMAD5 protein and mRNA in MC3T3-E1 cells under control media and the p-SMAD5 protein level during osteogenesis. After transfecting cells with the SMAD5 overexpression plasmids, the inhibitory effect of miR-155 on osteogenesis was significantly attenuated. In conclusion, miR-155 inhibited osteoblast differentiation by downregulating the translation of SMAD5 in mouse preosteoblast cells. Inhibition of miR-155 promoted osteogenic potential and thus it can be used as a potential target in the treatment of bone defects.

## 1. Introduction

Osteoblasts are the major cells involved in bone formation and development [1, 2]. Mature and functional osteoblasts are responsible for the synthesis, secretion, and mineralization of bone matrix, which is vital for bone strength and health [1, 2]. Therefore, a healthy osteogenic progression from preosteoblasts to osteoblasts is crucial in fracture healing as well as in bone defect regeneration. Current tissue regeneration techniques have provided new options for repairing large segmental bone defect, such as in vivo or ex vivo bone tissue engineering combining stem cells or preosteoblasts, tissue scaffolds, and biomaterials [3, 4]. Osteogenesis is a complex process [5–7], and enhanced understanding of its molecular pathways will benefit the clinical application of the bone tissue regeneration technique.

MicroRNAs (miRNAs) are a class of endogenously formed small RNA molecules that can combine with the 3′-untranslated region (3′-UTR) of a target gene to modulate gene expression at the posttranscriptional level [8]. A number of studies have confirmed that microRNAs play an important role in the regulation of osteogenesis. Wu et al. found that expression of the miR-30 family was downregulated in mouse preosteoblast differentiation and further found that miR-30 targeted the important transcription factors SMAD1 and RUNX2 [9]. Hassan et al. demonstrated that RUNX2 induced the expression of miR-23a/27a/24-2, which in turn acted as a negative feedback factor to hamper RUNX2 activity [10]. More recently, miR-155 was found to be downregulated during osteogenesis of human bone marrow mesenchymal stem cells [11]. However, the exact role and mechanism of miR-155 in osteogenesis remain unclear.

In this study, we aimed to elucidate the role of miR-155 during osteogenesis in the mouse preosteoblast cell line MC3T3-E1. More importantly, we also aimed to explore the potential downstream targets of miR-155.

## 2. Material and Methods

### 2.1. Cell Culture

The mouse preosteoblast cell line MC3T3-E1 was purchased from the Cell Culture Center, Institute of Basic Medical Sciences, Chinese Academy of Medical Sciences, School of Basic Medicine, Pecking Union Medical College (Beijing, China). HEK-293 cells were purchased from the American Type Culture Collection (ATCC, Manassas, VA, USA) for luciferase assay. Cells were routinely cultured in alpha-minimal essential medium (*α*-MEM; Gibco, Grand Island, NY, USA) supplemented with 100 units/mL penicillin and 100 *μ*g/mL streptomycin in a humid atmosphere with 5% CO_2_. The media were changed every 3 days. All procedures were approved by the Medical Ethic Committee of Jilin University, Changchun, China.

### 2.2. Osteogenic Induction

Osteogenesis was induced by adding 200 ng/mL bone morphogenetic protein 2 (BMP2) into the complete media for cell culture for 14 days [12]. Cells kept in normal media were used as the control.

### 2.3. Cell Transfection

MC3T3-E1 cells were transfected with miR-155 overexpression plasmids via the lentiviral transfection method. Briefly, 50 nM agomir-155 (Riobo, Guangzhou, China) or 50 nM antagomir-155 was added to the complete media for 48 hours. Cells transfected with miR-155-scramble were used as the transfection control. Differentiation of transfected cells was then induced with osteogenic media.

In addition, MC3T3-E1 cells were transfected with SMAD5-expressing lentiviruses or control lentiviruses (Hanheng, Shanghai, China) at a multiplicity of infection (MOI) of 10 in 5 mg/mL Polybrene (Sigma-Aldrich) for 12 h followed by treatment with 10 *μ*g/mL puromycin (Santa Cruz Biotechnology, CA, USA) for 21 days according to the manufacturer's protocol. Cells were then cotransfected with agomir-155 and cultured in osteogenic induction medium for 14 days.

### 2.4. Alkaline Phosphatase (ALP) Activity Assay

ALP activity was assessed at day 14 after osteogenic induction. MC3T3-E1 cells were firstly lysed using cell lysis buffer (Cell Signaling Technology, Boston, MA, USA) followed by centrifugation at 12,000*g* for 10 minutes. ALP activity was examined using an ALP activity kit according to the manufacturer's protocol (Beyotime, Shanghai, China) for 14 days after osteogenic induction. The absorbance was examined at 405 nm.

### 2.5. Alizarin Red Staining and Quantification

Alizarin Red staining was used to examine the calcification deposition in MC3T3-E1 cells 14 days after osteogenic induction. Cells were first fixed in neutral formalin buffer and washed with phosphate-buffered saline (PBS) three times. Ethanol (95%) was used for further dehydration, and cells were stained by Alizarin Red S solution for 1 minute. Cells were then soaked in acetone for 30 seconds followed by acetone-xylene 1 : 1 mixture solution for 15 seconds. The procedure was stopped once the red staining could be observed, and images were taken under a microscope.

The quantification of Alizarin Red staining was performed as described previously [13]. Briefly, Alizarin Red was extracted from the plates by incubation with 1 mL cetylpyridinium chloride buffer for 1 hour. Then the buffer was removed, and 200 *μ*L aliquots were transferred to a 96-well plate prior to reading at 550 nm. The levels of Alizarin Red and total protein were determined according to the standard curve, and the relative levels of Alizarin Red were represented as *μ*mol per *μ*g total protein.

### 2.6. Real-Time PCR

Total RNA of MC3T3-E1 cells was extracted on day 14 using the Trizol method (Sigma-Aldrich, MO, USA), and cDNAs were synthesized using EasyScript First-Strand cDNA Synthesis SuperMix (TransGen Biotech, Beijing, China). The RT-PCR system included 2.5 *μ*L in cDNA, 1 *μ*L of primer pair, and TransStart™ SYBR Green qPCR Supermix (TransGen Biotech, Beijing, China). The PCR cycle was set as 50°C for 2 min and 95°C for 10 min, followed by 40 cycles of 95°C for 15 sec, 60°C for 30 sec, and 72°C for 30 sec, with a final step at 72°C for 10 min. The miR-155 primers and the internal reference U6 were purchased from Guangzhou RiboBio Co., Ltd. (Guangzhou, China). The primers for SMAD5, ALP, and GAPDH were synthesized by Sangon Biotech, Shanghai, China. The primer sequences were as follows: SMAD5-F, CCAGCCGTGAAGCGATTG and SMAD5-F, GCCTTTTCTGCCCATTTCTCT; ALP-F, 5′-GAGCAGGAACAGAAGTTTGC-3′ and ALP-R, 5′-GTTGCAGGGTCTGGAGAGTA-3′; GAPDH-F, 5′-AACTCCCATTCCTCCACCTT-3′ and GAPDH-R, 5′-GAGGGCCTCTCTCTTGCTCT-3′. The expressions were calculated by 2^−ΔΔCT^ method using GAPDH expression level as internal control.

### 2.7. Western Blotting

MC3T3-E1 cells were lysed by cell lysis buffer containing the protease inhibitor PMSF (Cell Signaling Technology, MA, USA) and scratched on ice. The lysed buffer was centrifuged at 12000 r/min for 20 minutes, and the protein concentration in the supernatant was determined by BCA kit (Cell Signaling Technology, MA, USA). The protein samples were further prepared by mixing the same volume of loading buffer with 20 *μ*g of total protein that had been denatured under 100°C boiling water. The proteins were then separated on a 10% SDS-PAGE gel and transferred to a nitrocellulose blotting membrane under 200 mA for 60–90 minutes. The membrane was then incubated with primary rabbit anti-mouse SMAD5, p-SMAD5, or GAPDH antibody (1 : 1000; Abcam, Hongkang, China) under 4°C overnight after blocking with skim milk for 2 hours. Second goat anti-rabbit antibody conjugated with horseradish peroxidase (HRP) (1 : 5000; Abcam, Hongkang, China) was applied, and the membranes were incubated for 2 hours under room temperature. The protein banding was then determined using the enhanced chemiluminescence method (Cell Signaling Technology, MA, USA). The intensities of Western blot bands were determined, and protein expression was expressed as a ratio relative to GAPDH expression.

### 2.8. Luciferase Assay

The luciferase assay was conducted to demonstrate the binding site of miR-155 on Drosophila mothers against decapentaplegic protein 5 (SMAD5) in HEK-293 cells. Briefly, the 3′UTR of SMAD5 was amplified by PCR and inserted into the luciferase plasmids (SMAD5-3′UTR-wt). The SMAD5-3′UTR-mut was generated by point mutation of SMAD5-3′URT-wt. Plasmids with either 400 ng empty vector, 400 ng SMAD5-3′UTR-wt, or 400 ng SMAD5-3′UTR-mut were added to the cell culture for 48 hours. agomiR-155 and its scrambled control as well as 20 ng pRL-TK were also added to the cell culture. Luminescence intensity was then examined under luminometer (BioTek, VT, USA).

### 2.9. Statistical Analysis

All experiments were repeated at least three times. Data were analyzed using SPSS v.18.0 software (IBM SPSS, NY, USA). All data were presented as mean ± standard deviation (SD). Differences between two groups were identified by independent *t*-test, and one-way analysis of variance (ANOVA) was used to identify differences between more than three groups. A *P* value < 0.05 was considered statistically significant.

## 3. Results

### 3.1. miR-155 Expression is Reduced during Osteogenesis

**Figure 1:**
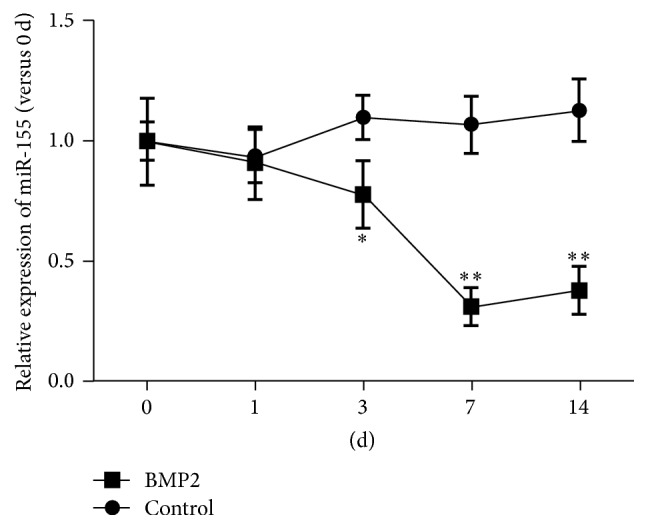
Expression of miR-155 during osteoblast differentiation of MC3T3-E1 cells. MC3T3-E1 cells were treated with 200 ng/mL BMP2 for osteogenic induction, and miR-155 expression was determined using qRT-PCR. Data are presented as mean ± SD; *n* = 6; ^*∗*^*P* < 0.05, ^*∗∗*^*P* < 0.01 compared with control.

During MC3T3-E1 osteogenic induction, the expression level of miR-155 was measured at different time points (Figure 1) using qRT-PCR. A significant trend of reduction was found over 14 days. Starting from day 3, the expression level of miR-155 was significantly less than that on day 0 (*P* < 0.05), and it was the lowest at day 7 (*P* < 0.01). At day 14, it was still significantly lower than that on day 0 (*P* < 0.01).

### 3.2. miR-155 Expression Attenuates Osteogenesis

**Figure 2:**
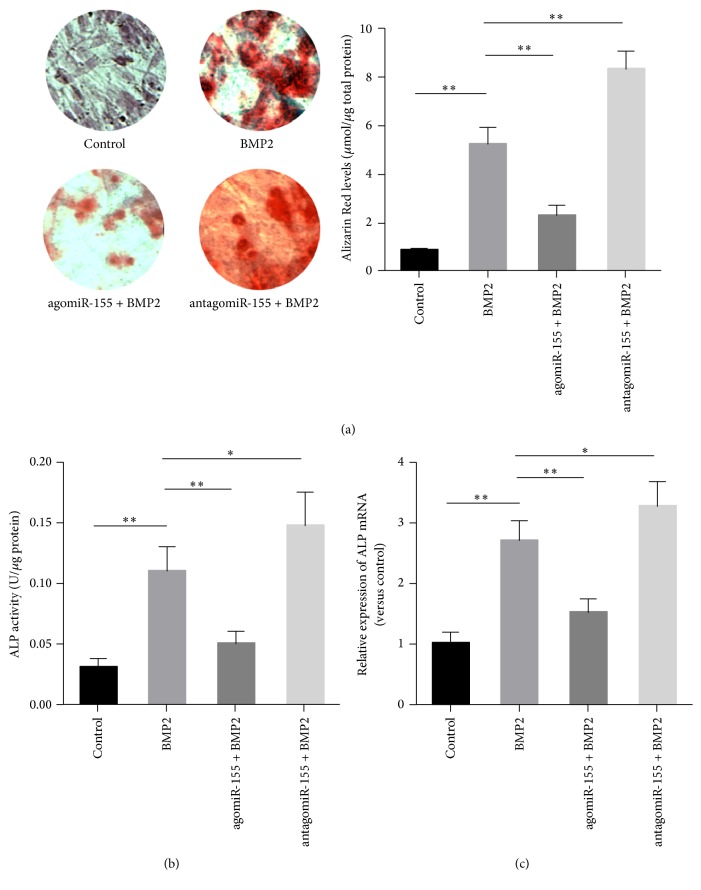
miR-155 modulated the osteoblast differentiation of MC3T3-E1 cells. MC3T3-E1 cells transfected with either agomiR-155 or antagomiR-155 were induced with 200 ng/mL BMP2 for 14 days. MC3T3-E1 cells left untreated (control) or only treated with BMP2 were used as the control and negative control groups, respectively. (a) Calcification deposition in MC3T3-E1 cells was determined using Alizarin Red staining. Alkaline phosphatase (ALP) activity (b) and mRNA level (c) were determined to quantify osteoblast differentiation of MC3T3-E1 cells. Data are presented as mean ± SD; *n* = 6; ^*∗*^*P* < 0.05 versus BMP2 group; ^*∗∗*^*P* < 0.01 versus control group or BMP2 group as indicated; BMP2: bone morphogenetic protein 2.

The osteogenic potential of MC3T3-E1 cells transfected with agomir-155 and antagomiR-155 was evaluated by ALP activity and calcification. As shown in Figure 2, cells undergoing osteogenesis showed significantly higher ALP activity (*P* < 0.01), ALP expression (*P* < 0.01), and calcification compared to cells cultured in control media (*P* < 0.01). Cells transfected with agomiR-155 had significantly lower levels of ALP activity (*P* < 0.01), ALP expression (*P* < 0.01), and Alizarin Red-stained calcification compared to the BMP2 group (*P* < 0.01). In contrast, cells transfected with antagomiR-155 (miR-155 inhibitor) showed significantly higher levels of ALP activity (*P* < 0.05), ALP expression (*P* < 0.05), and Alizarin Red-stained calcification compared to the BMP2 group (*P* < 0.01).

### 3.3. miR-155 Downregulates SMAD5 by Targeting Its 3′UTR

**Figure 3:**
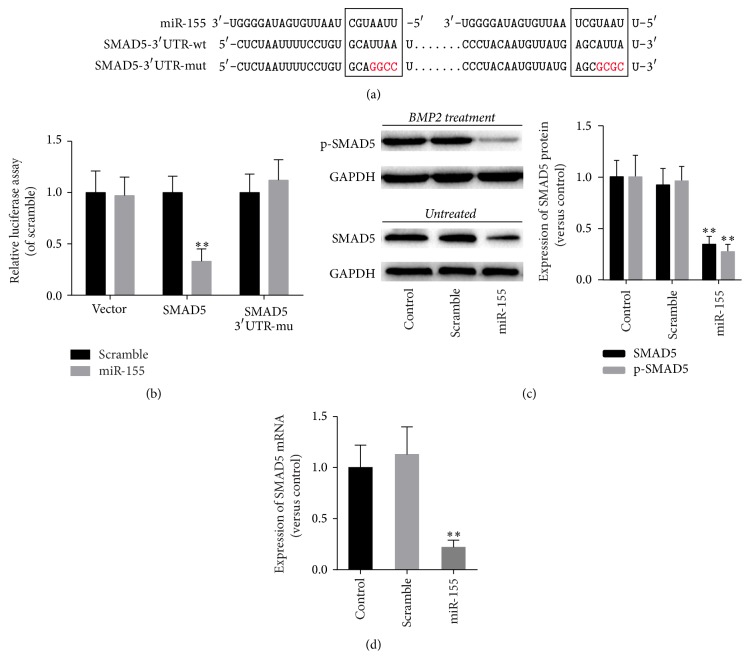
miR-155 downregulated SMAD5 expression by binding its 3′-UTR site. (a) Plasmid constructs of SMAD5 3′UTR-wt and SMAD5 3′UTR-mut containing either the wild-type or a mutant (marked with red) binding site sequence predicted from a database search; (b) miR-155 and SMAD5 plasmids or scramble control were cotransfected into HEK-293 cells and luciferase activities were determined; (c) SMAD5 protein was detected in MC3T3-E1 cells transfected with either miR-155 or scramble control in control media and p-SMAD5 protein levels were determined in MC3T3-E1 cells transfected with either miR-155 or scramble control after 30 minutes in osteogenic induction media; and (d) expression of SMAD5 mRNA in MC3T3-E1 cells transfected with either miR-155 or scramble control as determined by qRT-PCR; data are presented as mean ± SD; *n* = 6; ^*∗∗*^*P* < 0.05 versus scramble group.

To elucidate the mechanism of miR-155 in osteogenesis of preosteoblasts, we searched for the potential target sites of miR-155 in the TargetScan database. We found two potential binding sites in the 3′-UTR region of SMAD5 (Figure 3(a)). We then constructed a luciferase report system with SMAD5 mRNA 3′-UTR-wt and SMAD5 mRNA 3′-UTR-mu and transfected the HEK-293 cells with wild-type SMAD5 with agomiR-155, mutated SMAD5 with agomiR-155, vector control, or agomiR-155 scramble control. It was demonstrated that the wild-type SMAD5 group had a significantly lower luminescence intensity compared to the scramble control (*P* < 0.01), whereas the mutant SMAD5 group and the vector control group had similar luminescence intensities compared with the scramble group (*P* < 0.01; Figure 3(b)). These results indicated that miR-155 binds to the 3′UTR site of SMAD5 mRNA and begins translation.

We further investigated these effects of miR-155 in MC3T3-E1 cells by Western blotting. It was demonstrated that MC3T3-E1 cells transfected with agomiR-155 had significantly decreased p-SMAD5 protein expression during induced osteogenesis and SMAD5 protein expression in control media (both *P* < 0.01; Figure 3(c)). agomiR-155 also reduced SMAD5 mRNA levels in MC3T3-E1 cells treated with BMP2 for 30 minutes (*P* < 0.01; Figure 3(d)).

### 3.4. Overexpression of SMAD5 Alleviates the Inhibitory Effect of miR-155 on Osteogenesis

**Figure 4:**
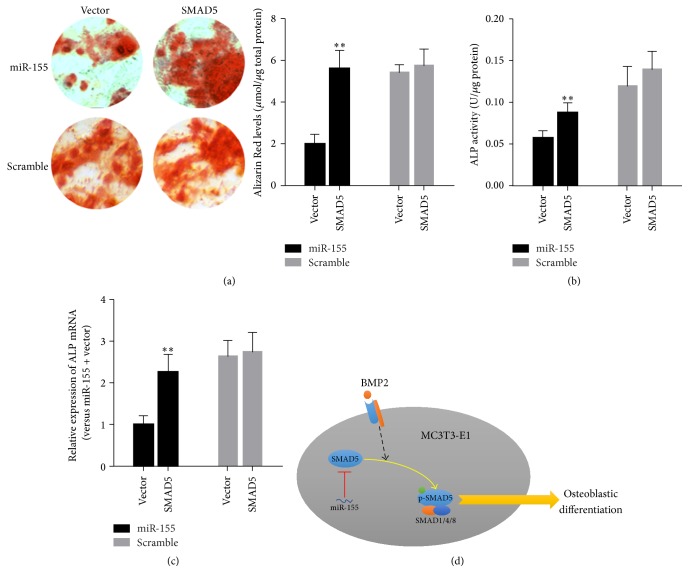
Overexpression of SMAD5 attenuated the inhibitory effect of miR-155 on osteoblastic differentiation. MC3T3-E1 cells transfected with SMAD5 lentiviral particles or the vector were cotransfected with either miR-155 or the scramble control and subsequently induced toward osteogenic differentiation. (a) Alizarin Red staining for calcification deposition. (b) Alkaline phosphatase (ALP) activity. (c) Relative mRNA expression of ALP. (d) A proposed model for the inhibitory effects of miR-155 on osteoblast differentiation in MC3T3-E1 cells. After BMP2 binds to the receptors, SMAD5 is directly activated via phosphorylation and forms a homomeric complex with SMAD1 and SMAD8. The heterooligomer translocates to the nucleus and then positively regulates the transcription of osteogenesis-related genes. miR-155 suppresses SMAD5 expression by directly targeting its mRNA and decreases the levels of p-SMAD5, thus leading to inhibition of osteoblastic differentiation of MC3T3-E1 cells. Data are presented as mean ± SD; *n* = 6; ^*∗∗*^*P* < 0.05 versus miR-155 + vector group.

To further investigate whether SMAD5 contributes to the effect of miR-155 on MC3T3-E1 differentiation, MC3T3-E1 cells transfected with SMAD5 lentiviral particles or empty vector as a negative control were cotransfected with agomiR-155 or the scramble control and subsequently treated with BMP2 (200 ng/mL) for 14 days. Cells treated with both agomiR-155 and SMAD5 showed higher ALP activity (*P* < 0.01), ALP expression (*P* < 0.01), and calcification deposition than cells transfected with agomiR-155 and empty vector (Figure 4).

## 4. Discussion

This study demonstrated the inhibitory role of miR-155 on the osteogenic potential of a mouse preosteoblast cell line. More importantly, miR-155 inhibited osteogenesis likely through binding to the 3′-UTR sites of SMAD5 mRNA, which hampered its translation. This was the first study demonstrating the role and signaling pathway of miR-155 during osteogenesis.

We demonstrated that miR-155 hampered osteogenesis of mouse preosteoblasts by decreasing ALP expression and activity and calcification, which is a characteristic of functional osteoblasts. Eguchi et al. demonstrated that the miR-155 expression level was reduced during osteogenesis of primary human osteoblasts [11], which was in accordance with the results of our study. The important role of miRNAs in cellular differentiation has been proposed recently. Previous research has suggested roles for different miRNAs during osteogenesis [11, 14]. On the other hand, the role of miR-155 in other cellular pathways has also been proposed. Chen et al. reported that miR-155 knockdown mice have an increased volume of brown fat via targeting at CCAAT/enhancer-binding protein *β* [15], indicating its role in adipogenesis and browning. miR-155 was also shown to suppress the differentiation of cardiomyocytes [16] and pose a negative effect on the differentiation of hematopoietic stem cells [17]. These data indicate that miR-155 has multiple biological functions in normal cellular differentiation, in addition to its first identified effect on tumorigenesis.

In this study, SMAD5 was found to be the target of miR-155 in osteogenesis of MC3T3-E1 cells. Transfection with agomiR-155 effectively reduced SMAD5 transcription. SMAD5 is an important downstream transcription factor for BMPs. Phosphorylation of SMAD5 caused by BMPs leads to the formation of a complex of SMAD5, SMAD1, and SMAD8, which further exerts the effect of BMPs after translocation into the nuclei [18, 19]. Previous studies have confirmed its important role in enhancing osteogenic potential [20, 21]. Based on our data with hampered mRNA and protein expression of both pSMAD5 and SMAD5 after overexpression of miR-155, the mechanism of miR-155's effect on SMAD5 was most likely through reducing the stability of SMAD5 mRNA. This inhibitory effect of miR-155 on SMAD5 was also found in primary rhesus macaque peripheral blood mononuclear cells with chronic simian immunodeficiency virus infection [22], a diffuse large B cell lymphoma cell line [23], and a lung epithelial cell line [24]. Furthermore, miR-155 was also demonstrated to inhibit a number of BMP signaling pathways including SMAD1, SMAD5, HIVEP2, CEBPB, RUNX2, and MYO10 [25]. Therefore, it remains possible that other targets of miR-155 may also play a part in its inhibitory role in osteogenesis. Taken together, our results indicate that miR-155 exerts its multiple biological functions on cellular development by interrupting SMAD5 translation.

In conclusion, this study demonstrated that miR-155 expression was reduced during osteogenesis induced by BMP2 in mouse preosteoblast cells. miR-155 hampered osteogenesis by binding to the 3′-UTR sites of SMAD5 mRNA and reducing its translation. Further studies are warranted to elucidate the exact role of miR-155 in other in vitro, animal, or human models. Inhibition of miR-155 promoted osteogenic potential and thus it can be used as a potential target in the treatment of large segmental bone defects.
